# The Electrophysiology of Disease Remission in Chronic Inflammatory Demyelinating Polyneuropathy

**DOI:** 10.1111/ene.70670

**Published:** 2026-06-24

**Authors:** Yusuf A. Rajabally, Joumana Freiha, Roshan Iqbal, David Allen, Chinar Osman

**Affiliations:** ^1^ Aston Medical School, Aston University Birmingham UK; ^2^ Inflammatory Neuropathy Service, Department of Neurology University Hospitals Birmingham Birmingham UK; ^3^ Wessex Neurological Centre, University Hospital Southampton Southampton UK

**Keywords:** chronic inflammatory demyelinating polyneuropathy, demyelinating, electrophysiology, remission, serial

## Abstract

**Background:**

Whether serial electrophysiology may be useful in chronic inflammatory demyelinating polyneuropathy (CIDP), in remission off treatment, is unknown.

**Methods:**

We retrospectively studied electrophysiological findings during active disease and post‐remission, in subjects with CIDP, at 2 UK neuropathy centres.

**Results:**

We included 24 consecutive subjects. Eighteen (75%) had typical CIDP, 3 (12.5%) motor CIDP and 3 (12.5%) distal CIDP. Mean age was 61.3 years (SD: 15.8). Mean interval between electrophysiological study during active disease and post‐remission was 63.25 months (SD: 52.0). Mean distal motor latency improved for median (*p* < 0.001), ulnar (*p* < 0.001), fibular (*p* = 0.01) and tibial (*p* = 0.002) nerves. Mean motor conduction velocity improved for median (*p* < 0.001), ulnar (*p* < 0.001) and fibular (*p* = 0.012) nerves. Mean minimum F‐wave latency improved for median (*p* = 0.003), ulnar (*p* = 0.002), and tibial (*p* = 0.027) nerves. The proportion of nerves showing conduction block was greater during active disease than post‐remission (26/66 vs. 10/67; *p* = 0.002). Criteria for demyelination were initially fulfilled by all subjects, but not post‐remission (24/24 vs. 14/24; *p* = 0.006). Electrophysiological quasi‐normalisation correlated with shorter pre‐treatment disease duration (*p* = 0.002). Amplitude of functional improvement correlated with fibular nerve motor amplitude at first study (*p* = 0.024).

**Conclusions:**

Electrophysiological quasi‐normalisation may occur in a third of subjects with typical CIDP in clinical remission off treatment and may be more likely with prompt diagnosis/treatment. Amelioration may be observed in all tested nerves. Initial fibular nerve motor amplitude may predict clinical improvement amplitude. The value of electrophysiology for prognostication/disease monitoring/management may merit further study in CIDP. Further research is warranted for subjects with variant and possible CIDP.

## Graphical abstract

Electrophysiological quasi‐normalisation may occur in a third of subjects with typical CIDP in clinical remission off treatment and may be more likely with prompt diagnosis/treatment. Electrophysiological amelioration may be observed in all tested nerves in CIDP in clinical remission off treatment. The value of electrophysiology for prognostication/disease monitoring/management may merit further study in CIDP.

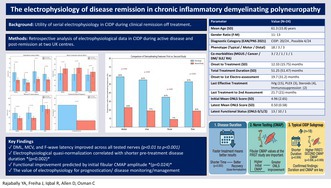

## 1 Introduction

Chronic inflammatory demyelinating polyneuropathy (CIDP) is a treatable disorder, with a proportion of affected subjects able to go into remission with or without residual functional deficits and with or without continuing treatment [1, 2].

Whether electrophysiology may be helpful as an objective and quantifiable accompaniment of clinical amelioration with treatment in CIDP in clinical practice remains unknown. Improvement of certain parameters has been documented in therapeutic studies [3], but provides no guidance for interpretation of serial electrophysiology in the context of clinical practice. In one study, the risk of clinical relapse after initial immunoglobulin response was found to be higher after treatment cessation in subjects with more demyelinating features [4]. Treatment withdrawal was in that analysis part of the study protocol, and disease remission, per se, was not considered. In another study comparing treatment‐dependent and treatment‐requiring subjects, no electrophysiological difference was identified, although only early electrophysiological data were considered. As a result, electrophysiological evolution with remission was not studied [5]. Similarly, a further study found that electrophysiology did not predict outcome in CIDP [6]. These studies did not investigate the potential value of serial electrophysiology in the context of clinical practice and in situations of disease remission after treatment withdrawal.

It is as such unknown whether disappearance or improvement of demyelinating features may be expected with treatment‐induced clinical amelioration, and if so, which parameters are the most likely useful. It is also unknown whether normalisation or quasi‐normalisation of the electrophysiological evaluation, as a whole, may occur with treatment. The value of electrophysiology in CIDP currently remains exclusively diagnostic in the absence of data demonstrating the value of serial studies [7]. The prognostic value of electrophysiology has been limited to mainly the ascertainment of evidence of axonal loss, which has been found to be associated with treatment‐unresponsiveness and worse outcomes [8]. Few studies have otherwise documented improvement of some electrophysiological parameters, but not others, immediately post‐immunoglobulin treatment in subjects on long‐term therapy [9].

In our units, electrophysiology is irregularly repeated in subjects with CIDP, while on treatment and after remission. We proposed to retrospectively study available electrophysiological results from our cohort, during active disease and treatment and compare the findings to those obtained after disease remission and off‐treatment. The main aims of this study were (i) to ascertain if demyelinating features improved across the two studies for most commonly evaluated parameters in routine clinical practice; (ii) to determine if electrophysiological normalisation or quasi‐normalisation may be observed in CIDP in remission off‐treatment; and (iii) to study the eventual correlations between clinical improvement with treatment with clinical and electrophysiological parameters.

## 2 Materials and Methods

We retrospectively reviewed records of subjects with a diagnosis of CIDP or possible CIDP, in current remission off treatment, meeting European Academy of Neurology/Peripheral Nerve Society (EAN/PNS) 2021 criteria [10], attending University Hospitals Birmingham and University Hospital Southampton, UK. Inclusion required (i) having been successfully treated with any immunomodulatory therapy; (ii) having been clinically stable through objective outcome‐measured evaluation for at least 6 months after treatment withdrawal; (iii) having reached “clinical remission”, defined as minimal persistent disability corresponding to an Overall Neuropathy Limitation Score (ONLS) ≤ 2, with a maximum of 1 point for each ONLS sub‐score; and (iv) having had electrophysiology performed during active and treated disease, and at any time after achieving remission and being taken off all treatments.

We collected demographics, disease sub‐type, mode of onset, pre‐treatment disease duration from symptom onset, pre‐treatment ONLS, and the latest achieved ONLS at remission maintained off‐treatment. The ONLS [11] is systematically utilised at clinic attendances in our practices (5 points for upper limb score, 7 points for lower limb score; optimal score of 0).

Median, ulnar, fibular and tibial motor nerves as well as median, ulnar, radial and sural sensory nerves were studied using standard equipment and procedures as described previously by specialist medical staff trained and qualified in electrophysiology [12]. Minimum electrophysiological study requirements were of 3 tested motor nerves and 2 tested sensory nerves. All available data were analysed using locally‐derived normative values and available recommendations for each electrophysiological parameter, as per availability of the detailed data at our centres, and as per the updated EAN/PNS Guidelines. We defined “electrophysiological quasi‐normalisation” as the change from fulfilment of EAN/PNS criteria for “CIDP” or “possible CIDP” at first electrophysiological evaluation to non‐fulfilment of the criteria for “CIDP” or “possible CIDP” at the second evaluation. In relation to local UK practice and consequentially, with the available data, we specifically analysed (i) distal motor latency (DML) for all 4 motor nerves, (ii) motor conduction velocity (MCV) for all nerves except the tibial nerve, (iii) minimum F‐wave latency for all nerves except the fibular. Presence of conduction block (CB) was ascertained outside compression sites up to Erb's point for median and ulnar nerves, and below the fibular neck for the fibular, as per EAN/PNS Guidelines. We did not include in the current analysis evaluation for distal compound muscle action potential duration prolongation and excessive proximal temporal dispersion, as these parameters are unfortunately rarely systematically studied quantitatively by neurophysiology staff in our laboratories. We, in addition, sought to ascertain (i) the clinical predictors of electrophysiological improvement and (ii) the electrophysiological predictors of functional clinical amelioration.

Statistical analyses were performed with SPSS 28.0 (Armonk, USA). To compare means or frequencies between groups, paired *t*‐tests or Fisher's Exact Tests were used, respectively. Correlations were studied through Spearman's Rank Correlations. Corrections for multiple correlations were not performed in view of the exploratory nature of this analysis. Independent associations were sought through logistic regression, including in models the co‐variables having demonstrated significant correlations. Significance was set at *p* < 0.05 for all tests.

This analysis was conducted as part of registered and approved retrospective clinical audits of the diagnosis and management of CIDP at University Hospitals Birmingham (CARMS‐20702) and University Hospital Southampton (SEV‐8371), UK. Audit does not require Ethics Committee approval in the UK.

## 3 Results

### 3.1 Study Subjects

Main results are summarised in Table 1. We included 24 subjects (12 subjects from Birmingham and 12 subjects from Southampton), meeting the above‐mentioned inclusion criteria for this study. There were 11 females and 13 males. Mean age was 61.3 years (SD 15.8). Twenty subjects (83.3%) had CIDP, and 4 subjects (16.7%) had possible CIDP, as per EAN/PNS criteria. Eighteen subjects (75%) had typical CIDP, 3 (12.5%) had motor CIDP and 3 (12.5%) had distal CIDP. In terms of co‐morbidities, one subject had type 2 diabetes, two had IgM kappa monoclonal gammopathy of uncertain significance (MGUS) without anti‐MAG antibodies, one had IgG kappa MGUS, one had systemic lupus erythematosus, one had rheumatoid arthritis and 2 had treated prostatic cancer. Mean pre‐treatment disease duration was 12.33 months (SD: 15.75). Mean total duration of treatment with any therapeutic agent was 51.25 months (SD: 51.47). Mean total pre‐treatment ONLS was 4.96 (SD: 2.65) and mean final achieved ONLS at remission was 0.50 (SD: 0.58). Remission without sequelae (ONLS = 0) was achieved by 13 subjects (54.2%), 10 subjects (41.7%) had a final ONLS of 1, and 1 subject (4.2%) had a final ONLS of 2. Latest administered effective and withdrawn treatment, prior to diagnosis of remission was immunoglobulin in 15 subjects (62.5%), plasma exchange in 3 (12.5%), pulse corticosteroids in 4 (16.7%), and immunosuppression in 2 (8.3%).

**TABLE 1:** Characteristics of 24 consecutive subjects with a diagnosis of CIDP in remission off treatment, studied with serial electrophysiology, from Birmingham, UK and Southampton, UK.

Mean age‐years (SD)	61.3 (15.8)
Gender F:M (ratio)	11:13
CIDP diagnostic category, as per EAN/PNS, 2021	CIDP: 20/24
	Possible CIDP: 4/24
CIDP phenotype	Typical: 18/24
	Motor: 3/24
	Distal: 3/24
Co‐morbidities	Diabetes: 1/24
	MGUS: 3/24
	SLE:1/24
	RA:1/24
	Treated prostatic cancer:2/24
Mean pre‐treatment disease duration‐months (SD)	12.33 (15.75)
Mean total duration of treatment‐months (SD)	51.25 (51.47)
Mean initial total ONLS (SD)	4.96 (2.65)
Mean latest ONLS (SD)	0.50 (0.58)
ONLS scores at latest assessment: breakdown	ONLS = 0: 13/24
	ONLS = 1: 10/24
	ONLS = 2: 1/24
Last effective treatment administered	Immunoglobulin: 15/24
	Plasma exchange: 3/24
	Corticosteroids: 4/24
	Immunosuppressant: 2/24
Mean interval between 2 electrophysiological assessments‐months (SD)	63.25 (52)
Mean interval between disease onset and first electrophysiological assessment‐months (SD)	19.7 (31.2)
Mean interval between last treatment administered and second electrophysiological assessment‐months (SD)	21.7 (21.0)

All subjects had undergone electrophysiology (i) during active disease and while on treatment, and (ii) after remission and off treatment. Mean interval between the 2 electrophysiological assessments was 63.25 months (SD: 52). Mean interval between disease onset and the first electrophysiological evaluation was 19.7 months (SD: 31.2). Mean interval between the administration of the last treatment for CIDP and the second electrophysiological evaluation was 21.7 months (SD: 21.0).

### 3.2 Electrophysiological Findings in the Full Cohort

Twenty subjects (83.3%) met EAN/PNS 2021 criteria for CIDP at the first study, compared to 12 (50%) at the second study (*p* = 0.031). All 24 subjects met criteria for CIDP or possible CIDP at the first study, whereas only 14 subjects (58.3%) did at the second study (*p* = 0.0006). Hence, electrophysiological quasi‐normalisation occurred in 10 subjects (41.7%), but complete normalisation (all parameters returning within normal range) was not observed in any subject.

The individual motor nerve analysis for fulfilment of demyelinating criteria across the 2 studies is shown in Table 2. The median nerve showed demyelinating features in 47 of 80 tested nerves (58.75%) at first study vs. 21 of 92 tested nerves (22.8%) at second study (*p* < 0.001). The ulnar nerve showed demyelinating features in 44 of 80 tested nerves (55%) at first study vs. 17 of 89 tested nerves (19.1%) at second study (*p* < 0.001). The fibular nerve showed demyelinating features in 22 of 63 tested nerves (34.9%) at first study vs. 5 of 55 tested nerves (9.1%) at second study (*p* < 0.001). The tibial nerve showed demyelinating features in 18 of 34 tested nerves (52.9%) at first study vs. 7 of 37 tested nerves (18.9%) at second study (*p* = 0.003).

**TABLE 2:** Individual motor nerve analysis of presence of demyelinating features in 24 consecutive subjects with a diagnosis of CIDP in remission off treatment, studied with serial electrophysiology, from Birmingham, UK and Southampton, UK.

Nerve	Proportion of nerves presenting with demyelinating features at first study	Proportion of nerves presenting with demyelinating features at second study	Significance (*p*)
Median	47/80	21/92	*p* < 0.001
Ulnar	44/80	17/89	*p* < 0.001
Fibular	22/63	5/55	*p* < 0.001
Tibial	18/34	7/37	*p* = 0.003

Comparative results for individual parameters are shown in Table 3. Compound muscle action potential amplitudes improved for median and ulnar nerves across the 2 studies (*p* = 0.002 and *p* < 0.001, respectively), but not for the fibular or tibial nerves (*p* = 0.24 and *p* = 0.16, respectively). Distal motor latency (DML) improved for median, ulnar, fibular and tibial nerves (*p* < 0.001, p < 0.001, *p* = 0.01 and *p* = 0.002, respectively). Motor conduction velocity improved for median, ulnar and fibular nerves (*p* < 0.001, p < 0.001 and *p* = 0.012, respectively). Minimum F‐wave latency improved for median, ulnar and tibial nerves (*p* = 0.003, *p* = 0.002 and *p* = 0.027, respectively). The proportion of tested nerves showing conduction block (CB) was reduced across the 2 studies (26/66 at initial evaluation vs. 10/67 at second evaluation; *p* = 0.002).

**TABLE 3:** Per parameter, per nerve analysis of presence of demyelinating features in 24 consecutive subjects with a diagnosis of CIDP in remission off treatment, studied with serial electrophysiology, from Birmingham, UK and Southampton, UK.

Parameter/nerve	First Study done during active disease (mean (SD), or proportion)	Second Study done after remission (mean (SD), or proportion)	Significance (*p*)
Distal motor latency/median nerve	8.0 (3.7)	4.7 (1.1)	*p* < 0.001
Distal motor latency/ulnar nerve	5.2 (2.2)	3.6 (1.1)	*p* < 0.001
Distal motor latency/fibular nerve	8.4 (4.9)	5.1 (1.0)	*p* = 0.01
Distal motor latency/tibial nerve	7.3 (2.7)	4.9 (0.9)	*p* = 0.002
Motor conduction velocity/median nerve	32.9 (12.6)	41.8 (9.2)	*p* < 0.001
Motor conduction velocity/ulnar nerve	31.3 (11.2)	43.2 (9.4)	*p* < 0.001
Motor conduction velocity/fibular nerve	30.2 (10.7)	35.6 (8.8)	*p* = 0.012
Minimum F‐wave latency/median nerve	53.1 (16.6)	37.0 (6.9)	*p* = 0.003
Minimum F‐wave latency/ulnar nerve	54.1 (18.6)	37.7 (6.4)	*p* = 0.002
Minimum F‐wave latency/tibial nerve	78.1 (19.4)	65.6 (13.1)	*p* = 0.027
Conduction block/median, ulnar (up to Erb's point); Fibular (below fibular neck)	26/66	10/67	*p* = 0.002

No changes of sensory nerve action potential (SNAP) amplitudes were ascertained across the 2 studies for the sural nerve (*p* = 0.75). An improvement of the mean radial SNAP was observed at the second evaluation compared to the first; however, not reaching significance (*p* = 0.06).

### 3.3 Electrophysiological Findings in the Subset of 18 Subjects With Typical CIDP

Seventeen of 18 (94.4%) subjects with typical CIDP met EAN/PNS 2021 criteria for CIDP at the first study, compared to 10/18 (55.6%) at the second study (*p* = 0.018). All 18 subjects with typical CIDP met criteria for CIDP or possible CIDP at the first study, whereas only 11/18 (61.1%) did at the second study (*p* = 0.008). Hence, electrophysiological quasi‐normalisation was observed in 7 subjects (38.9%).

With regards to individual motor nerve analyses in the typical CIDP subgroup, the median nerve showed demyelinating features in 36 of 62 tested nerves (58.1%) at first study vs. 19 of 69 tested nerves (27.5%) at the second study (*p* < 0.001). The ulnar nerve showed demyelinating features in 36 of 62 tested nerves (58.1%) at first study vs. 14 of 68 tested nerves (20.6%) at the second study (*p* < 0.001). The fibular nerve showed demyelinating features in 21 of 52 tested nerves (40.4%) at first study vs. 5 of 47 tested nerves (10.6%) at the second study (*p* = 0.0011). The tibial nerve showed demyelinating features in 14 of 26 tested nerves (53.8%) at first study vs. 6 of 27 tested nerves (22.2%) at the second study (*p* = 0.01).

Compound muscle action potential amplitudes in the typical CIDP subgroup improved for median and nerves across the 2 studies (*p* = 0.009 and *p* < 0.001, respectively), but not for fibular or tibial nerves (*p* = 0.32 and *p* = 0.28, respectively). DML improved for median, ulnar, fibular and tibial nerves (*p* = 0.002, *p* < 0.001, *p* = 0.02 and *p* = 0.006, respectively). Motor conduction velocity improved for median, ulnar and fibular nerves (*p* < 0.001, *p* < 0.001 and *p* = 0.0114, respectively). Minimum F‐wave latency improved for median and ulnar nerves (*p* = 0.004 and *p* = 0.008) but not for the tibial nerve (*p* = 0.11). The proportion of nerves showing CB was reduced across the 2 studies (23/51 at first evaluation vs. 8/53 at second evaluation; *p* = 0.0012).

No changes in sural SNAP amplitudes were ascertained in between the 2 studies (*p* = 0.46). A difference, again not reaching significance, was observed for the radial SNAP amplitudes (*p* = 0.066).

### 3.4 Correlation Studies Between Clinical and Electrophysiological Parameters in the Full Cohort

Electrophysiological quasi‐normalisation correlated only with shorter pre‐treatment disease duration (*p* = 0.002), but not with age, gender, pre‐treatment ONLS, ONLS at remission, ONLS improvement amplitude, interval between disease onset to initial electrophysiological study, or interval between last treatment administered to second electrophysiological study.

Correlation analyses were also performed between (i) ONLS improvement amplitude and (ii) complete remission (ONLS = 0), with all studied electrophysiological variables. ONLS improvement amplitude correlated only with fibular nerve CMAP amplitude at the first study (*p* = 0.024). Complete remission was not associated with any electrophysiological parameter at first evaluation and correlated only with fibular CMAP amplitude at the second study (*p* = 0.009).

### 3.5 Correlation Studies Between Clinical and Electrophysiological Parameters in the Typical CIDP Subgroup

In the typical CIDP subgroup, electrophysiological quasi‐normalisation correlated, as in the full cohort, exclusively with shorter pre‐disease duration (*p* = 0.008). However, ONLS improvement amplitude correlated with both (i) fibular nerve CMAP amplitude at the first study (*p* = 0.006), and (ii) fibular nerve CMAP amplitude at the second study (*p* = 0.009). Complete remission was, on the other hand, not associated with any electrophysiological parameter.

## 4 Discussion

The electrophysiological outcome is uncertain, in subjects with treated CIDP having attained clinical remission. Electrophysiology, widely available and used for decades for CIDP diagnosis, has not to date been described as a useful biomarker of disease activity. Few studies have considered the electrophysiological progression in subjects with CIDP in therapeutic trials [3, 13, 14]. These demonstrated improvement correlating with clinical improvement, although the specific question of what may be found in circumstances of remission without continuing treatment was not addressed. On the other hand, few studies have exclusively considered the value of initial electrophysiology for prognostication [4, 5, 6]. The wide variation of electrophysiological practice in inflammatory neuropathy, as shown in Guillain‐Barré syndrome [15], may be partly contributory to this, as may also be the lack of awareness and the adequate application of guidelines detailing best electrophysiological practice [16, 17, 18]. Whether electrophysiological quasi‐normalisation may occur and be helpful for disease monitoring is unknown, and whether amelioration of demyelinating features is to be expected with treatment has remained uncertain.

In the current retrospective study, we compared electrophysiological assessments in subjects who had gone into clinical remission with treatment and remained in remission following treatment withdrawal. We included, for consistency, subjects with a final post‐treatment ONLS ≤ 2, who had been successfully taken off treatment, and who had subsequently remained in remission for at least 6 months. We found that electrophysiological quasi‐normalisation, as defined here, occurred in > 40% of subjects after clinical remission. Complete electrophysiological normalisation was not observed. These findings indicate that total reversal of the features of demyelination (which may include abnormal values, however not attaining EAN/PNS cut‐offs for demyelination) is not uncommon in treated subjects with CIDP attaining remission. Although management decisions should primarily be based on functional progression, we believe this may be helpful in clinical practice in multiple possible real‐life situations, including uncertainty about the organicity of severe on‐going deficits despite treatment, doubt about efficacy of a utilised therapy, or discrepancy in between serial strength and functional evaluations using different outcome measures. Of note, none of the subjects eligible for this analysis had focal/multifocal CIDP, which may suggest less likely disease remission in this variant, as indicated previously through their described possible greater refractoriness to treatment compared with typical CIDP [19].

All tested motor nerves and all tested parameters for demyelination improved across the 2 studies. This is in keeping with resolution of inflammatory demyelination as the driver of functional impairment in CIDP. However, axonal loss as determined by CMAP amplitudes improved exclusively in the upper limbs but not in the lower limbs. This may be consistent with the length dependent axonopathy occurring secondarily to inflammatory demyelination in CIDP [20], rendering less likely treatment‐induced recovery of axonal loss in the longest nerves. Interestingly, in keeping with previously described possible preferential involvement of radial sensory nerves compared to sural nerves in CIDP [21, 22], we found a trend for improvement of radial SNAPs but not of sural SNAPs at remission.

We found that electrophysiological quasi‐normalisation was associated only with pre‐disease treatment duration. Of relevance, we have previously found a similar association of pre‐disease treatment duration with likelihood of treatment withdrawal, as well as with better prognosis, in other recent studies [23, 24]. These findings all globally support early diagnosis and treatment as a modifiable factor of long‐term outcome in CIDP. The amplitude of functional improvement was otherwise only associated with the initial fibular nerve CMAP amplitude, which therefore represented the only potential predictor for the extent of clinical amelioration. This is consistent with previous studies, which indicated poor treatment response to immunoglobulin in subjects with lower fibular or summated CMAPs at baseline [8, 25]. As may be expected, complete clinical remission was associated with fibular CMAP amplitude at the follow‐up study, indicating better preserved axonal function in these subjects, whereas no electrophysiological predictors could be ascertained from the first electrophysiological study. This finding is similarly comparable to those of previous studies, which demonstrated association of motor strength and function with CMAP amplitudes, in a cohort of subjects stabilised on treatment [20, 26].

Our study has several limitations. The design was retrospective and the number of subjects included was small, due to non‐systematic and, as a result, irregular performance of serial electrophysiology in our practices, as well as to the inclusion criteria requiring at second assessment, clinical stability for 6 months, minimal residual disability and treatment discontinuation. The repeat electrophysiology had been performed as a result of patient or physician concern or curiosity about objective electrophysiological evolution or presence of subjective non‐specific symptoms not suggestive of clinical relapse, such as fatigue or transient sensory symptoms. As a result, inclusion in the current analysis may have been subject to selection bias. Phenotypically, our cohort was heterogeneous although we additionally performed analyses exclusively on subjects with typical CIDP, which did not reveal substantially different findings, considering the reduced sample size. The study did not include a control group of subjects who had not achieved remission, which may have provided interesting additional comparative data. Also, we included four subjects with possible CIDP, for whom the concept of quasi‐normalisation may not be as meaningful, as these subjects had demyelinating changes at initial study in only a single nerve. Caution is therefore appropriate with the concept of quasi‐normalisation of electrophysiology, especially in subjects with possible CIDP. Finally, we considered a single outcome measure, the ONLS, as had been routinely administered in all cases at all out‐patient clinic visits, in both our centres. Electrophysiological studies were also not done in a systematic or protocolized manner. The evaluation of potentially important parameters such as distal and proximal dispersion, which are reported as of common occurrence in CIDP [27, 28], could unfortunately not be performed. Finally, the timing of both initial and follow‐up electrophysiological studies was variable, although it did accurately correspond to the 2 stages studied: period of disease activity versus period of clinical stability off‐treatment.

Despite these limitations, we believe our study may offer novel insights and support further research to determine if electrophysiology may be a relevant biomarker of disease activity in CIDP. Larger prospective studies are needed to better define the role of electrophysiological measures in CIDP, particularly within therapeutic trials of emerging agents, where such assessments have recently been underrepresented in study design [29, 30]. Reintegrating electrophysiological outcomes into both clinical research and routine practice may be especially relevant in the context of novel therapies with proposed protective effects on synaptic and axonal function [31, 32].

## Author Contributions

**Yusuf A. Rajabally:** conceptualization, investigation, methodology, writing – original draft, project administration, supervision, formal analysis. **Joumana Freiha:** investigation, methodology, data curation, formal analysis. **David Allen:** methodology, data curation, validation. **Roshan Iqbal:** methodology, investigation, validation. **Chinar Osman:** investigation, writing – original draft, validation, methodology, supervision.

## Funding

The authors have nothing to report.

## Conflicts of Interest

Y.A.R. has received consultancy honoraria from Sanofi, Argenx, Janssen, LFB, Polyneuron, Grifols, Takeda, Dianthus, Vitaccess; has received educational sponsorships from LFB and CSL Behring; has obtained research grants from LFB. CO has received speaker/consultancy honoraria from Takeda, Grifols and Terumo BCT. JF, R.I. and D.A. have no disclosures.

## Data Availability

The data that support the findings of this study are available on request from the corresponding author. The data are not publicly available due to privacy or ethical restrictions.
